# Consumption of Nuts at Midlife and Healthy Aging in Women

**DOI:** 10.1155/2020/5651737

**Published:** 2020-01-07

**Authors:** Tania-Marisa Freitas-Simoes, Maude Wagner, Cecilia Samieri, Aleix Sala-Vila, Francine Grodstein

**Affiliations:** ^1^Lipid Clinic, Department of Endocrinology and Nutrition, Hospital Clínic de Barcelona, IDIBAPS, Barcelona, Spain; ^2^Ciber Fisiopatología de la Obesidad y Nutrición (CIBEROBN), Instituto de Salud Carlos III (ISCIII), Madrid, Spain; ^3^Univ. Bordeaux, Inserm, Bordeaux Population Health Research Center, UMR U1219, F-33000 Bordeaux, France; ^4^Channing Division of Network Medicine, Department of Medicine, Brigham and Women's Hospital, Boston, MA, USA

## Abstract

**Background:**

Nut consumption may reduce age-related diseases and lead to better health and well-being in aging. Many conditions of aging develop over decades, and thus earlier lifestyle factors may particularly influence later health.

**Methods:**

In 1998 and 2002, we administered food frequency questionnaires to assess nut consumption (peanuts, walnuts, and other nuts and peanut butter) in women in the Nurses' Health Study in their 50 s/early 60 s. In 2012, those who survived beyond 65 years with no chronic diseases, no reported memory impairment, no physical disabilities, and intact mental health were considered “healthy agers.” We used multivariable logistic regression to estimate odds ratios for healthy versus usual aging, controlled for sociodemographic, behavioral, dietary, and other potential confounding factors.

**Results:**

Of 33,931 participants at midlife, 16% became “healthy agers.” After age adjustment, we observed a significant association between total nut consumption at midlife and higher odds of healthy aging, with strongest associations observed excluding peanut butter (odds ratio (OR) = 1.46, 95% confidence interval (CI) 1.32–1.62, ≥3 servings/week versus none). Findings were attenuated after further control for covariates, including overall diet quality (OR = 1.14, 95% CI 1.02–1.28, *P* trend = 0.05). For nut types, we found statistically significantly higher odds of healthy aging across peanuts, walnuts, and other nuts after age adjustment. After full control for confounders, only walnut consumption remained associated with healthy aging (*P* trend = 0.0001); for example, the OR was 1.20 (95% CI 1.00–1.44) for ≥2 servings/week versus none.

**Conclusions:**

Women consuming nuts at midlife have a greater likelihood of overall health and well-being at older ages. Nut consumption may represent a simple intervention to explore and promote healthy aging.

## 1. Introduction

A public health priority is to identify strategies to promote healthy aging [1]. However, there is abundant evidence of links between diet and individual age-related diseases [2, 3] and premature mortality [4], yet population data on diet and overall health and well-being in aging are limited. Such knowledge could contribute to health/prevention guidelines that are broadly applicable across a range of health conditions. In our own previous research in the Nurses' Health Study (NHS), we reported that better diet quality [5, 6] and a greater dietary flavonoid intake [7] at midlife were related to a greater likelihood of overall health and well-being in aging, defined as longevity with no major chronic diseases, good mental health, and no impairments in either cognitive or physical function.

More specifically, observational epidemiologic studies and randomized controlled trials provide evidence supporting the association of regular nut consumption with decreased risk in cardiovascular disease and all-cause [8–10] mortality. Consistent with this evidence on vascular disease, nut consumption was incorporated into the 2013 ACC/AHA lifestyle management guidelines [11]. In addition to the primary prevention of cardiovascular diseases, there is increasing epidemiologic evidence that nut consumption is related to better cognitive health [12–14], and initial studies suggest that nuts are related to better physical function in aging as well [15]. However, to our knowledge, the issue of whether long-term nut consumption relates to healthy aging as a broader concept remains unexplored.

Thus, we utilized the NHS to examine consumption of nuts at midlife and likelihood of subsequent healthy aging in women; as women live longer than men on average, understanding healthy aging in women is particularly important.

## 2. Materials and Methods

### 2.1. Nurses' Health Study

The NHS is a prospective cohort study that began in 1976, when female nurses aged 30 to 55 years from 11 US states completed mailed questionnaires. Follow-up questionnaires every two years update information about health and lifestyle; follow-up remains approximately 90%.

In 1980, participants completed a semiquantitative food frequency questionnaire (FFQ) [16], which was repeated approximately every 4 years thereafter; starting in 1998, more detailed questions on intake of nuts were added. In 1992 and every four years thereafter, the Medical Outcomes Study Short Form-36 (SF-36) was administered, which evaluates eight health concepts including physical functioning [17]. In 2012, questions to address subjective memory concerns were included. This study was approved by the Institutional Review Board of Brigham and Women's Hospital (Boston, MA) (protocol 1999P011114).

To determine the population for analysis in the present study, we used 1998–2002 as the study baseline, corresponding to the years when detailed data on nut consumption were first available. We focused on this relatively early time period for baseline, since we were concerned that, at older ages, underlying health could influence diet choices and lead to bias in analyses. Moreover, scientifically, many chronic diseases and health conditions take years to develop, and thus, it is biologically likely that risk factors at earlier timepoints are most important to later health. We defined healthy aging from the 2012 follow-up questionnaire, which was the first time we collected information simultaneously on chronic diseases, memory concerns, physical function, and mental health from the full cohort. Among the 55,318 nurses who responded to the questions on healthy aging in 2012, we excluded 13,353 women who had already been diagnosed with one of the 11 major chronic diseases of interest (see list below) at study baseline and 7,479 women who did not provide baseline dietary data in 1998 and 2002, as well as 555 women who were missing adequate data to calculate diet quality (AHEI score). Thus, 33,931 participants were included in analyses.

### 2.2. Assessment of Nut Consumption

To assess nut consumption at midlife, we averaged nut intake from the FFQs in 1998 and 2002, when more detailed information on nuts was available; thus, 1998–2002 represented our baseline here. Averaging diet over several timepoints provides a measure of longer-term intake (which is likely most relevant to chronic diseases and conditions) and also reduces variability of the measurement. We separately asked participants how often they had consumed (1) peanuts, (2) walnuts, and (3) other nuts (serving size 28 g (1 oz)) during the preceding year: never or almost never, 1 to 3 times a month, once a week, 2 to 4 times a week, 5 or 6 times a week, once a day, 2 or 3 times a day, 4 to 6 times a day, or more than 6 times a day. Consumption of peanut butter was assessed separately, with the same 9 responses (serving size 15 g (1 tablespoon)). Total nut consumption was the sum of peanuts, walnuts, other nuts, and peanut butter. We also examined total nut consumption excluding peanut butter, since peanut butter can contain added oils, such as hydrogenated fats.

### 2.3. Ascertainment of Healthy Aging

To separate “healthy” from “usual” aging, we considered 4 health domains, all measured in 2012. We considered as “healthy” agers women who survived beyond 65 years of age, with no history of chronic diseases, no reported memory impairment, no physical disabilities, and intact mental health; remaining women who survived but did not achieve good health in one or more domains were “usual” agers [6]. The definitions of “healthy” for each domain are provided below.

For the chronic disease domain, we considered history of 11 chronic diseases, reported by women on the biennial questionnaires [18]. The diseases included primary causes of death in the US and several additional debilitating diseases: cancer (other than nonmelanoma skin cancer), myocardial infarction, coronary artery bypass surgery or percutaneous transluminal coronary angioplasty, congestive heart failure, stroke, type 2 diabetes, kidney failure, chronic obstructive pulmonary disease, Parkinson's disease, multiple sclerosis, and amyotrophic lateral sclerosis.

The memory domain was assessed with 7 questions (yes/no) to assess memory concerns [19]. The questions inquired about participants' perception of their change in ability to remember things, trouble remembering recent events, trouble remembering short lists, trouble remembering things from one second to the next, spoken instructions, trouble following conversations or plot on TV, and trouble finding their way on familiar streets. Healthy for this domain was defined as at most one memory concern. Strong relations have been found between subjective memory concerns and performance on objective neuropsychologic tests [20, 21], including in a subset of our cohort with both subjective and objective data, and as well as for imaging and neuropathologic markers in other cohorts [22].

Impairment of physical function was assessed by the SF-36 [23] physical function items. This inquires about physical limitations in performing the following activities: moderate activities (e.g., moving a table, pushing a vacuum cleaner, bowling, and playing golf); bathing and dressing yourself; walking 1 block; walking several blocks; walking 1 mile; vigorous activities (e.g., running, lifting heavy objects, and strenuous sports); bending, kneeling, or stooping; climbing 1 flight of stairs; climbing several flights of stairs; and lifting or carrying groceries. Each question had 3 response choices: “Yes, limited a lot,” “Yes, limited a little,” or “No, not limited at all.” No impairment of physical function was defined as having no limitations on moderate activities and no more than moderate limitations on vigorous activities.

Mental health was assessed through the Geriatric Depression Scale with 15 items (GDS-15) [24]. Good mental health was considered as a GDS-15 score less than or equal to 4, the median in our population.

### 2.4. Assessment of Covariates

Sociodemographic, lifestyle, and health-related covariates (age, educational level, marital status, multivitamin use, aspirin use, smoking, and energy intake) were obtained from the biennial questionnaires; median income was determined from census tract data. All covariates were ascertained in 1998, except the educational level and marital status, which were only available in 1992 and 1996, respectively. Body mass index (BMI) was calculated as weight in kilograms divided by height in meters squared. Physical activity was assessed every 2–4 years by using validated Nurses' Health Study Physical Activity Questionnaire [25]. We calculated the Alternative Healthy Eating Index-2010 (AHEI-2010), to assess overall diet quality [26]. This score includes 11 components: high intakes of vegetables (excluding potatoes), fruits (excluding juices), whole grains, nuts, and legumes; long-chain omega-3 polyunsaturated fatty acids (*n*−3 PUFA); PUFA (excluding long-chain *n* − 3 PUFA); and lower intakes of sugar-sweetened beverages and fruit juice; red and processed meats; trans fat; and sodium. Each AHEI-2010 component is scored from 0 (worst) to 10 (best) according to component-specific criteria. In addition, moderate alcohol intake of 1/2 to 1.5 drinks per day is assigned the “best” score, with lower scores for excess (≥2.5 drinks per day) or nondrinking. For our analysis, we excluded the component “nuts and legumes,” given that was our primary study variable; thus, AHEI-2010 scores were based on 10 components and ranged from 0 (nonadherence) to 100 (perfect adherence).

### 2.5. Statistical Analysis

We assessed nut consumption (1998–2002) when nurses were in their late 50 s and early 60 s. To investigate the relation between nut consumption at midlife and healthy aging, we used age-adjusted and multivariable-adjusted logistic regression models. We categorized participants into 5 groups according to frequency of consumption of (i) total nuts and (ii) total nuts excluding peanut butter: never or <1 per month, 1–<4 per month, 1–<2 per week, 2–<3 per week, and ≥3 per week and into 4 groups according to consumption of walnuts, peanuts, other nuts and peanut butter: never or <1 per month, 1–<4 per month, 1–<2 per week, and ≥2 per week. The first logistic regression model was adjusted for age (years), and a second model for age and sociodemographic, lifestyle, and health-related covariates, including education (registered nurse, bachelor's degree, master, or doctorate), marital status (widowed, married, and single/separated/divorced), median income from census tract (quintiles), BMI (<22, 22–24.9, 25–29.9, and ≥30), energy intake (quintiles of kcal/day), multivitamin use (yes/no), aspirin use (<1, 1–2, or >2 tablets/week), pack-years of smoking (quintiles), and physical activity (quintiles of MET-h/week). The third model was further adjusted for diet quality (AHEI-2010 score, excluding the component of nuts; quintiles).

We conducted several secondary analyses. In one, we slightly revised the “usual aging” definition to add 1,302 women who died during follow-up (i.e., between baseline and 2012), resulting in a reference group of 29,695 rather than 28,393 women. In addition, we conducted analyses separately examining each healthy aging domain (e.g., any chronic disease versus none) in logistic models. All *P* values were two-sided. Ninety-five percent confidence intervals (95% CI) were calculated for odds ratios (ORs). Analyses were performed using SAS version 9.1 (SAS Institute). The Nurses' Health Study data used to generate the results in this manuscript are available upon request. Procedures to access data are described at https://www.nurseshealthstudy.org/researchers (contact e-mail: nhsaccess@channing.harvard.edu).

## 3. Results

Of 33,931 women, 5,538 (16%) were considered “healthy” agers; the remaining 28,393 (84%) were “usual” agers (data not shown in table). There was a mix of factors which led to classification as usual aging; that is, one-third of women with usual aging had one or more chronic diseases, and over half had a limitation in functional domains of aging (memory function, mental health, and physical function).

We compared characteristics of healthy versus usual agers at study baseline (Table 1). Women who were healthy agers in later life were younger (mean age = 58 vs 62 years, respectively), with somewhat higher levels of education than usual agers (41% vs 32% had bachelor's degree or higher, respectively). Women who achieved healthy aging had a lower prevalence of overweight and obesity at baseline than usual agers (39% vs 58%, respectively) and fewer pack-years of smoking (mean pack-years = 7 versus 11, respectively) at midlife. They also reported higher levels of physical activity (mean = 26 versus 18 METs/week) than usual agers. Finally, a history of high blood pressure was less prevalent at baseline in women who achieved healthy aging than usual aging (21% vs 39%, respectively), as was a history of high cholesterol (39% vs 56%, respectively).

When we examined the relation of nut consumption at midlife and subsequent odds of healthy aging (Table 2), we observed a significant association between total nut intake and higher odds of healthy aging, for analyses including and excluding peanut butter, in age-adjusted models (*P* trend < 0.0001 for both). Findings were not as strong when we included peanut butter (OR = 1.18, 95% CI 1.04–1.34 for ≥3 servings/week versus no intake of total nuts); in contrast, when we excluded peanut butter, this OR was 1.46 (95% CI 1.32–1.62). After further controlling for covariates, including diet quality, the association for total nuts with peanut butter was attenuated and borderline statistically significant (OR = 1.10, 95% CI 0.96–1.26 for ≥3 servings/week, *P* trend = 0.160). For total nuts without peanut butter, we continued to observe significantly greater odds of healthy aging, with 3 or more servings per week, after control for all covariates (OR = 1.14, 95% CI 1.02–1.28, *P* trend = 0.046).

We also considered each type of nut (peanuts, peanut butter, walnuts, and other nuts) (Table 3). In age-adjusted models, we found statistically significantly better odds of healthy aging across peanuts (*P* trend = 0.01), walnuts (*P* trend < 0.0001), and other nuts (*P* trend < 0.0001). After control for all potential confounding factors, especially overall diet quality, all results were attenuated. Walnut consumption alone remained associated with statistically significantly better odds of healthy aging (OR = 1.20, 95% CI 1.00–1.44 for ≥2 servings per week versus no intake, *P* trend = 0.0001).

In secondary analyses (data not shown in table), on adding women who died during follow-up to the “usual aging” group, the results remained highly similar; for example, for total nut intake, the odds ratio for 3 + servings/week versus <1/month remained 1.10 and the overall *P* trend was 0.09, and for total nuts excluding peanut butter, this odds ratio was 1.15 with an overall *P* trend = 0.03. In addition, when we separately examined nut intake in relation to each healthy aging domain, we generally found that nut intake was related to apparently better odds of healthy aging across most domains. For example, for total nuts, comparing 3 + servings/week to <1/month, odds of good health in each domain ranged from 1.05 to 1.10 for physical function, mental health, and chronic disease history (and odds of good health was worse only for memory complaints, with OR = 0.84); for the four individual domains, the overall *P* trends were borderline or not statistically significant.

## 4. Discussion

In this large cohort of women, we observed a significant association between consumption of nuts at midlife and healthy aging, broadly defined across four domains—chronic diseases, mental health, and cognitive and physical function. When analyzing several specific types of nuts, walnut consumption appeared to have the strongest relation with healthy aging. Importantly, our findings that nut consumption is associated with broad-based health in aging is particularly relevant to public health and suggests that small dietary changes have potential as simple and relatively inexpensive approaches to promote health and well-being in aging.

Our findings are consistent with scientific literature supporting cardiometabolic benefits of nuts [27], as well as benefits for brain health [12, 28, 29]. Moreover, our findings may be explained biologically by the nutrient composition of nuts. Nuts are energy and nutrient-dense foods, rich in fat (in particular unsaturated fatty acids) but also with a notable content of proteins, vitamins, nonsodium minerals, and fiber. In addition, they have an array of bioactive phytochemicals, primarily polyphenols [30]. The unique composition of nuts may act synergistically in reducing oxidation and inflammation, features at the core of the aging process [27]. In addition, a plausible explanation for the robustness in associations observed for walnuts is their differential composition compared to other nuts, namely, the presence of *α*-linolenic acid (a plant omega-3 fatty acid) and the higher content of phytosterols and polyphenols, in particular ellagitannins [31].

Our study has limitations. First, in this observational study, confounding is a limitation; however, we considered a broad array of potential confounding factors, including overall diet quality. In addition, the homogeneity of our population of nurses reduces confounding by many factors (such as health consciousness and healthcare access) and helps to provide strong internal validity. Third, nut intake was self-reported and some measurement errors are inevitable. However, we administered a validated FFQ to assess nut consumption, and averaged consumption over two reports, four years apart, which decreases variability; moreover, it is most likely that this error was random; thus, the error would have led to bias to the null or an underestimate of relations between nuts and healthy aging.

Our study has strengths, including a large sample size, the long duration of follow-up, the high follow-up, and the comprehensive, multidomain evaluation of healthy aging. Importantly, the long follow-up period allowed us to evaluate nut consumption at midlife, a critical period of initiation and development for many aging conditions.

## 5. Conclusion

In summary, we found that consumption of nuts at midlife was related to a greater likelihood of overall health and well-being at older ages. The association was particularly robust for walnuts, a source of alpha-linolenic acid and ellagitannins. Since many health conditions of aging develop over decades and, thus, earlier lifestyle factors likely have the most influence on later health, our results support the notion that long-term consumption of nuts, a fairly low-cost dietary intervention, merits further confirmation as a strategy contributing to healthier lifespan.

## Figures and Tables

**Table 1:** Characteristics at study baseline^a^ of usual agers and healthy agers in the Nurses' Health Study.

Characteristics^b^	Healthy agers (*n* = 5,538)	Usual agers (*n* = 28,393)
Mean age, years	58 (4.9)	62 (6.5)

Educational level (1992), % (*n*)		
Registered nurse	59 (3267)	68 (19307)
Bachelor's degree	25 (1385)	21 (5963)
Master or doctorate	16 (886)	11 (3123)

Marital status (1996), % (*n*)		
Widowed	5 (277)	10 (2839)
Married	86 (4763)	82 (23282)
Separated/divorced	9 (498)	8 (2271)

Median neighborhood income, $	69,517 (27,170)	64,929 (24,843)

Body mass index, % (*n*)		
<22	26 (1440)	16 (4543)
22–24.9	35 (1938)	27 (7666)
25–29.9	31 (1717)	36 (10221)
≥30	8 (443)	22 (6246)

Mean alternate healthy eating index-2010 score (excluding nuts)	47 (9)	45 (9)

Mean alcohol intake, g/day	6 (8)	5 (9)

Multivitamin use, % (*n*)	61 (3378)	62 (17604)

Aspirin use, tablets per week, % (*n*)		
<1	68 (3766)	62 (17604)
1‐2	7 (388)	7 (1988)
>2	25 (1384)	31 (8802)

Pack-years of smoking	7 (12)	11 (17)

Mean physical activity, metabolic equivalent task-hours/week	26 (27)	18 (21)

History of high blood pressure, % (*n*)	21 (1163)	39 (11073)

History of high cholesterol, % (*n*)	39 (4984)	56 (15900)

^a^Study baseline is 1998 for this table, unless otherwise noted. Measures in table were calculated among nonmissing values (≤5% of data were missing). ^b^Data expressed as mean (SD) or as percent.

**Table 2:** Odds of healthy aging, according to frequency of total consumption of nuts at midlife in the Nurses' Health Study.

	Servings (28 grams for nuts and 15 grams for peanut butter)					
	<1/month	1–<4/month	1–<2/week	2–<3/week	≥3/week	*P* trend
Total nuts						
Healthy agers (*n* = 5,538), %	6.6	21.6	21.6	14.8	35.3	
Age-adjusted OR (95% CIs) of healthy vs usual aging	1.0 (Ref.)	1.02 (0.90, 1.17)	1.02 (0.89, 1.16)	1.05 (0.91, 1.21)	1.18 (1.04, 1.34)	<0.0001
Multivariable-adjusted^a^ OR (95% CIs) of healthy vs usual aging	1.0 (Ref.)	1.07 (0.93, 1.24)	1.04 (0.90, 1.20)	1.08 (0.93, 1.25)	1.14 (1.00, 1.31)	0.031
Multivariable-adjusted^b^ OR (95% CIs) of healthy vs usual aging	1.0 (Ref.)	1.06 (0.92, 1.22)	1.02 (0.88, 1.17)	1.05 (0.90, 1.22)	1.10 (0.96, 1.26)	0.160

Total nuts excluding peanut butter						
Healthy agers (*n* = 5,538), %	20.8	35.2	20.2	8.2	15.6	
Age-adjusted OR (95% CIs) of healthy vs usual aging	1.0 (Ref.)	1.12 (1.03, 1.22)	1.17 (1.07, 1.29)	1.32 (1.17, 1.49)	1.46 (1.32, 1.62)	<0.0001
Multivariable-adjusted^a^ OR (95% CIs) of healthy vs usual aging	1.0 (Ref.)	1.10 (1.01, 1.20)	1.08 (0.97, 1.19)	1.14 (1.00, 1.31)	1.21 (1.09, 1.35)	0.001
Multivariable-adjusted^b^ OR (95% CIs) of healthy vs usual aging	1.0 (Ref.)	1.08 (0.99, 1.18)	1.04 (0.94, 1.15)	1.09 (0.95, 1.25)	1.14 (1.02, 1.28)	0.046

^a^Logistic regression model adjusted for age (years), education in 1992 (registered nurse, bachelor's degree, and master or doctorate), marital status in 1996 (widowed, married, and single/separated/divorced), census tract median income (quintiles), BMI (kg/m^2^; <22, 22–24.9, 25–29.9, and ≥30), energy intake (quintiles of kcal/day), multivitamin use (yes/no), aspirin use (<1, 1‐2, or >2 tablets/week), pack-years of smoking (quintiles), physical activity (quintiles of METs-h/week), and physical function impairment in 2000 (yes/no). All covariates were collected in 1998 unless stated otherwise. ^b^Logistic regression model further adjusted for Alternative Healthy Eating Index-2010 score (quintiles), excluding nuts as a component.

**Table 3:** Odds of healthy aging, according to frequencies of consumption of specific types of nuts at midlife in the Nurses' Health Study.

	Servings (28 grams for nuts)				
	<1 per month	1–<4 per month	1–<2 per week	≥2 per week	*P* trend
Peanuts					
Healthy agers (*n* = 5,538), %	39.2	45.6	7.4	7.9	
Age-adjusted OR (95% CIs) of healthy vs usual aging	1.0 (Ref.)	1.01 (0.94, 1.07)	1.05 (0.93, 1.18)	1.20 (1.06, 1.35)	0.011
Multivariable-adjusted^a^ OR (95% CIs) of healthy vs usual aging	1.0 (Ref.)	1.00 (0.93, 1.07)	0.96 (0.85, 1.10)	1.08 (0.95, 1.22)	0.524
Multivariable-adjusted^b^ OR (95% CIs) of healthy vs usual aging	1.0 (Ref.)	0.99 (0.92, 1.06)	0.94 (0.83, 1.07)	1.04 (0.92, 1.19)	0.960

Peanut butter					
Healthy agers (*n* = 5,538), %	21.5	38.8	14.6	25.2	
Age-adjusted OR (95% CIs) of healthy vs usual aging	1.0 (Ref.)	0.95 (0.88, 1.03)	0.94 (0.85, 1.04)	0.94 (0.86, 1.03)	0.220
Multivariable-adjusted^2^ OR (95% CIs) of healthy vs usual aging	1.0 (Ref.)	1.00 (0.92, 1.09)	1.01 (0.90, 1.12)	1.00 (0.91, 1.10)	0.946
Multivariable-adjusted^3^ OR (95% CIs) of healthy vs usual aging	1.0 (Ref.)	1.00 (0.92, 1.09)	1.00 (0.90, 1.12)	0.99 (0.90, 1.09)	0.839

Walnuts					
Healthy agers (*n* = 5,538), %	51.8	40.1	4.6	3.5	
Age-adjusted OR (95% CIs) of healthy vs usual aging	1.0 (Ref.)	1.25 (1.17, 1.33)	1.49 (1.29, 1.73)	1.63 (1.38, 1.93)	<0.0001
Multivariable-adjusted^a^ OR (95% CIs) of healthy vs usual aging	1.0 (Ref.)	1.17 (1.10, 1.26)	1.25 (1.07, 1.47)	1.28 (1.07, 1.53)	<0.0001
Multivariable-adjusted^b^ OR (95% CIs) of healthy vs usual aging	1.0 (Ref.)	1.14 (1.07, 1.22)	1.19 (1.01, 1.39)	1.20 (1.00, 1.44)	0.0001

Other nuts					
Healthy agers (*n* = 5,538), %	47.0	39.1	7.3	6.6	
Age-adjusted OR (95% CIs) of healthy vs usual aging	1.0 (Ref.)	1.11 (1.04, 1.19)	1.37 (1.22, 1.55)	1.35 (1.19, 1.53)	<0.0001
Multivariable-adjusted^a^ OR (95% CIs) of healthy vs usual aging	1.0 (Ref.)	1.05 (0.98, 1.13)	1.19 (1.04, 1.36)	1.09 (0.95, 1.25)	0.018
Multivariable-adjusted^b^ OR (95% CIs) of healthy vs usual aging	1.0 (Ref.)	1.03 (0.96, 1.10)	1.14 (1.00, 1.30)	1.03 (0.90, 1.18)	0.205

^a^Logistic regression model adjusted for age (years), education in 1992 (registered nurse, bachelor's degree, master, or doctorate), marital status in 1996 (widowed, married, and single/separated/divorced), census tract median income tract (quintiles), BMI (kg/m^2^; <22, 22–24.9, 25–29.9, and ≥30), energy intake (quintiles), multivitamin use (yes/no), aspirin use (<1, 1–2, or >2 tablets/week), pack-years of smoking (quintiles), physical activity (quintiles of METs-h/week), and physical function impairment in 2000 (yes/no). All covariates were collected in 1998 unless stated otherwise. ^b^Logistic regression model further adjusted for Alternative Healthy Eating Index-2010 score (quintiles) excluding the component of nuts.

## Data Availability

The Nurses' Health Study data used to generate the results in this manuscript are available upon request. Procedures to access data are described at https://www.nurseshealthstudy.org/researchers (contact e-mail: nhsaccess@channing.harvard.edu).
